# Computational Study of Photodegradation Process and Conversion Products of the Antidepressant Citalopram in Water

**DOI:** 10.3390/molecules28124620

**Published:** 2023-06-07

**Authors:** Yifan Shen, Se Wang, Ying Lu, Kai Chen, Li Luo, Ce Hao

**Affiliations:** 1 Collaborative Innovation Center of Atmospheric Environment and Equipment Technology, Jiangsu Key Laboratory of Atmospheric Environment Monitoring and Pollution Control, School of Environmental Science and Engineering, Nanjing University of Information Science and Technology, Nanjing 210044, China; 20201248128@nuist.edu.cn (Y.S.);; 2 School of Chemistry and Life Sciences, Suzhou University of Science and Technology, Suzhou 215009, China; 3 State Key Laboratory of Fine Chemicals, School of Chemical Engineering, Dalian University of Technology, Dalian 116024, China

**Keywords:** citalopram, photodegradation, conversion products, time-dependent density functional theory, density functional theory

## Abstract

Citalopram (CIT) is a commonly prescribed medication for depression. However, the photodegradation mechanism of CIT has not yet been fully analyzed. Therefore, the photodegradation process of CIT in water is studied by density functional theory and time-dependent density functional theory. The calculated results show that during the indirect photodegradation process, the indirect photodegradation of CIT with ·OH occurs via OH-addition and F-substitution. The minimum activation energy of C10 site was 0.4 kcal/mol. All OH-addition and F-substitution reactions are exothermic. The reaction of ^1^O_2_ with CIT includes the substitution of ^1^O_2_ for F and an addition reaction at the C14 site. The *E*_a_ value of this process is 1.7 kcal/mol, which is the lowest activation energy required for the reaction of ^1^O_2_ with CIT. C–C/C–N/C–F cleavage is involved in the direct photodegradation process. In the direct photodegradation of CIT, the activation energy of the C7-C16 cleavage reaction was the lowest, which was 12.5 kcal/mol. Analysis of the *E*_a_ values found that OH-addition and F-substitution, the substitution of ^1^O_2_ for F and addition at the C14 site, as well as the cleavage reactions of C6–F/C7–C16/C17–C18/C18–N/C19–N/C20–N are the main pathways of photodegradation of CIT.

## 1. Introduction

Depression is a common disease in modern life, which has a particular impact on people’s daily life quality, and a large number of suicides are caused by depression [1,2,3]. By 2014, more than 350 million people had suffered from depression, according to global studies [1]. Since the emergence of COVID-19, the global ripple effects have put more people at risk for depression by putting them out of work [4,5,6].

Antidepressants are especially used to treat depression and other related diseases. The gradual increase in patients with depression has led to an increasing demand for such drugs. According to an OECD survey, antidepressant use doubled in several OECD countries from 2000 to 2017 [7]. The widespread use of antidepressants worldwide can lead to their being released into the environment and causing a range of environmental problems. These antidepressant drugs released into the environment are widely present in environmental water bodies, which will affect the ecological environment of aquatic organisms to a certain extent and may cause harm to them and have a negative impact on the surrounding environment [8]. Some antidepressant residues have been found in wastewater [8,9,10].

Citalopram (CIT) is an effective antidepressant to treat depression and related disorders. This drug in the human body after metabolism is discharged into wastewater, and then it is released after being treated by the sewage treatment station [9,10,11,12,13]. CIT was measured in effluent concentrations of 11–322 ng/L, and based on the current research, it has been found that some antidepressants are widely present in environmental water during the study of the degradation process of antidepressants [9,10,11,13]; at the same time, the concentrations measured downstream of the sewage treatment station were 40–90 ng/L [9,12]. In the current research process, antidepressants have various forms in water bodies in the natural environment, and the general treatment process cannot completely and effectively remove them, so there will be a wide range of residues in surface water during the degradation process, and the residual concentration of CIT was about 1327 ng/L in Canadian wastewater. CIT residues of 110 ng/L were found in Texas waters [12]. Activated sludge helps reduce environmental damage from residues in water bodies [14].

Therefore, it can reduce the harm of these residual drugs in the water body to the aquatic environment to a certain extent. Thus, after the treatment process of general sewage treatment plants, due to the role of activated sludge, the sewage entering the sewage treatment plant can be effectively degraded [14]. However, the adsorption effect of CIT in activated sludge is not satisfactory [15]. It can be seen that the general treatment process cannot effectively solve the problem of these residues in water, and the residual antidepressants in water will cause harm to organisms, even at low concentrations [16,17,18]. In the treatment of these antidepressants, the commonly used treatment methods have no significant effect on the removal of antidepressants in water and have a significant water detection rate. These residual antidepressants will have a negative impact on aquatic organisms in water and water quality in residual areas [16,17,18].

Therefore, it is necessary to study the degradation of CIT in environmental water to evaluate the harm to the ecological environment caused by the degradation process, and in recent years, the photodegradation process has been proven to be an effective way to remove some antidepressants from water. Many studies have explored the degradation pathway of antidepressants in water and the structure of products after photodegradation in water [19,20,21,22,23,24,25,26]. CIT in natural water can also be effectively removed by photodegradation [25]. The photodegradation process can be divided into two types. In the process of direct photodegradation, a CIT molecule absorbs a photon and generates an excited CIT molecule, then a photochemical reaction occurs. During indirect photodegradation, CIT reacts with certain substances present in water, such as hydroxyl radicals (·OH) and singlet oxygen molecules (^1^O_2_), which are produced by dissolved organic matter, nitrate, and nitrite involved in the photochemical reaction [27]. The photodegradation process can remove CIT from water, so it is of great significance to determine the photodegradation process in water. It was found that the ·OH-addition reaction and ·OH-substitution of F atom reaction of CIT occur in the indirect photodegradation process [28]. The study found the material structure after the reaction of C and N [29]. The above studies show that CIT undergoes photodegradation in water, and many intermediate products will be produced during the degradation process. However, no other studies have found the structures and corresponding properties of these intermediates and whether the corresponding derivatives will be transformed into more toxic intermediates during the photochemical reaction, which will have a more negative impact on the environment. These problems require further research and exploration. In the photodegradation process of CIT, the existing research can only determine part of the products and their structures after photodegradation. The corresponding reaction mechanism and reaction path cannot be pointed out, and the main degradation path and corresponding product structure in water cannot be determined. Therefore, exploring its mechanism in the photodegradation process in water can help to grasp the detailed information of the photodegradation process of CIT and provide effective information for environmental protection and corresponding environmental pollution problems and environmental risk assessment.

Density functional theory (DFT) and time-dependent density functional theory (TDDFT) are increasingly used to study chemical properties and photochemical transformation pathways of substances [30,31,32,33,34,35,36,37]. In this study, DFT and TDDTT were used to study the photochemical transformation process and reaction types of CIT in a water environment.

In the previous studies of CIT, it can be found that most of the studies described the photodegradation pathway of CIT in the water environment through actual experimental operations and speculated the degradation pathway of CIT based on the product structure detected during the experiment. However, there is no clear explanation of the photodegradation reaction pathway of CIT and the structure and corresponding photochemical properties of the byproducts in the photodegradation process. At the same time, the main product structure and the corresponding main reaction pathway in the photodegradation process of CIT cannot be explained during the experiment. However, the corresponding theoretical calculation research can explain the process and mechanism of the reaction from the molecular level, which can be combined with the previous experimental conclusions of CIT research to verify the possible degradation pathway of the reactants and the structure of the degradation products. At the same time, the study on the photodegradation of CIT at the molecular level can obtain the structure and corresponding photochemical properties of the byproducts in the degradation process. On this basis, the reaction degradation pathway in the photodegradation process of CIT can be further speculated. The method of DFT and TDDFT can explain the reaction mechanism of CIT in the direct and indirect photodegradation processes and the reaction type in the reaction process from the molecular point of view [31,32,33,34,35,36,37]. The activation energy in the reaction process can explain the main reaction pathway in the reaction process, which provides a theoretical basis for the conclusion of the experimental study and reveals the structure of more byproducts that may exist in the experimental process but are not obtained. Therefore, in this study, the photolysis process of CIT in a water environment was studied by theoretical calculation.

## 2. Results and Discussion

### 2.1. CIT Optimized Geometry

**Figure 1:**
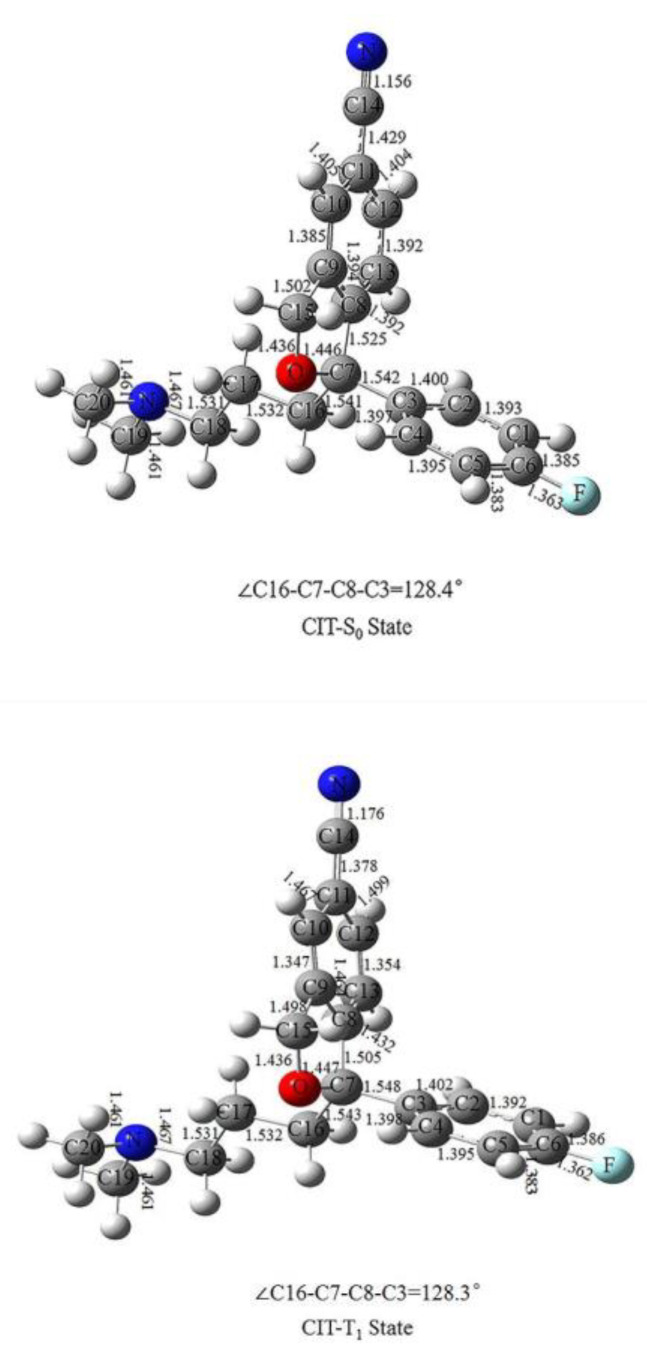
Geometry optimization in the ground state (S0) and in the excited state (T1) of CIT with selected bond lengths (Å) and dihedral angles (°).

The optimized geometries of CIT in the ground state (S_0_) and T_1_ state are shown in Figure 1. In the benzene ring, the length of bond at C2–C3 in the S_0_ state is 1.400 Å, which is 0.002Å shorter than that in the T_1_ state. There is no significant difference in the bond lengths between other atoms, the differences being in the range of 0.001 Å. The bond length of C6–F is 1.362 Å in the T_1_ state and 1.363 Å in the S_0_ state. The C3–C7 length of bond is 1.542 Å in the S_0_ state, which is 0.006 Å shorter than in the T_1_ state, indicating that the benzene ring is easier to remove in the T_1_ state. In the C8–C13, there is a noticeable difference in the corresponding bond lengths between the S_0_ and the T_1_ state; the C8–C9 length of bond is 1.394 Å in the S_0_ state, which is 0.07 Å shorter than that in the T_1_ state. The C9–C10 bond length is 1.347 Å in the T_1_ state, which is 0.038 Å shorter than that in the S_0_ state. The C10–C11 length of bond is 1.405 Å in the S_0_ state, which is 0.062 Å shorter than that in the T_1_ state. The C11–C12 length of bond is 1.404 Å in the S_0_ state, which is 0.095 Å shorter than that in the T_1_ state. The C12–C13 length of bond is 1.354 Å in the T_1_ state, which is 0.038 Å shorter than that in the S_0_ state. The C8–C13 length of bond is 1.392 Å in the S_0_ state, which is 0.04 Å shorter than that in the T_1_ state. The C7–C8 bond length is 1.505 Å in the T_1_ state, which is 0.02 Å shorter than that in the S_0_ state. The C9–C15 length of bond is 1.498 Å in the T_1_ state, which is 0.004 Å shorter than that in the S_0_ state. The length of bond at C7–O and C15–O are not much different in the T_1_ and S_0_ states. The bond lengths corresponding to C16–C20, N–C20, and N–C19 remain the same in the T_1_ and S_0_ states.

### 2.2. Indirect Photodegradation of CIT via ·OH in Water

**Figure 2:**
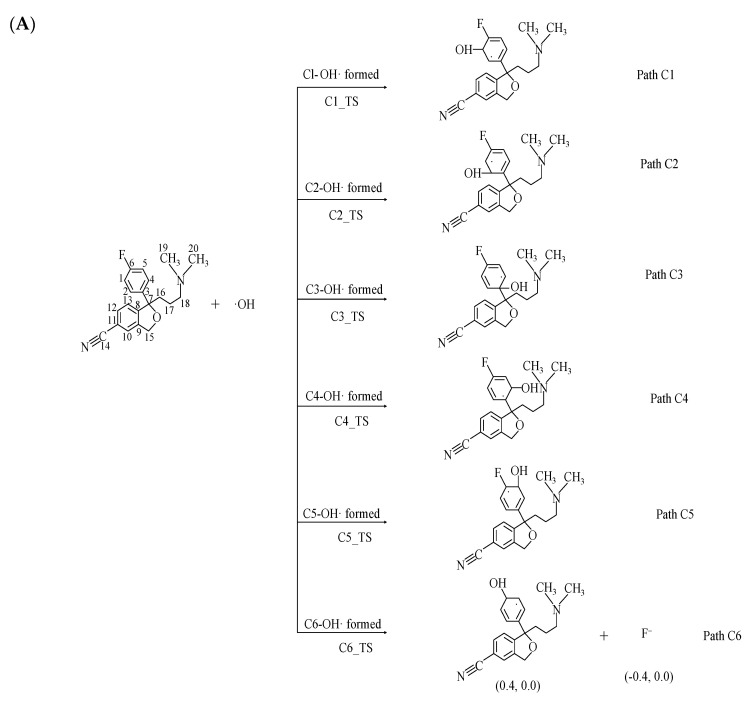

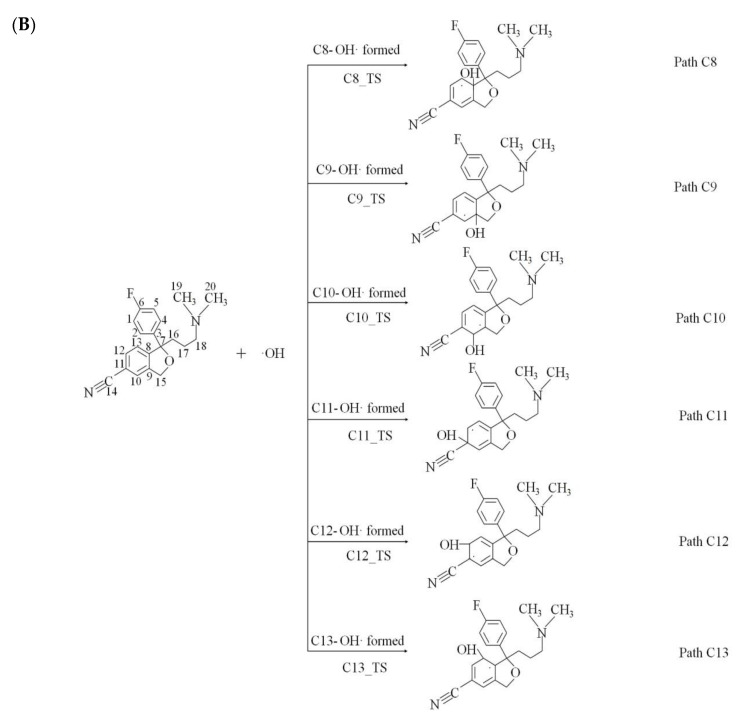
Indirect photodegradation pathway of CIT and ·OH at the C1–C6/C8–C13 sites. The figures in parentheses are NBO charge and electron spin density of products (NBO charge, electron spin density): (**A**) C1–C6 sites. (**B**) C8–C13 sites.

**Figure 3:**
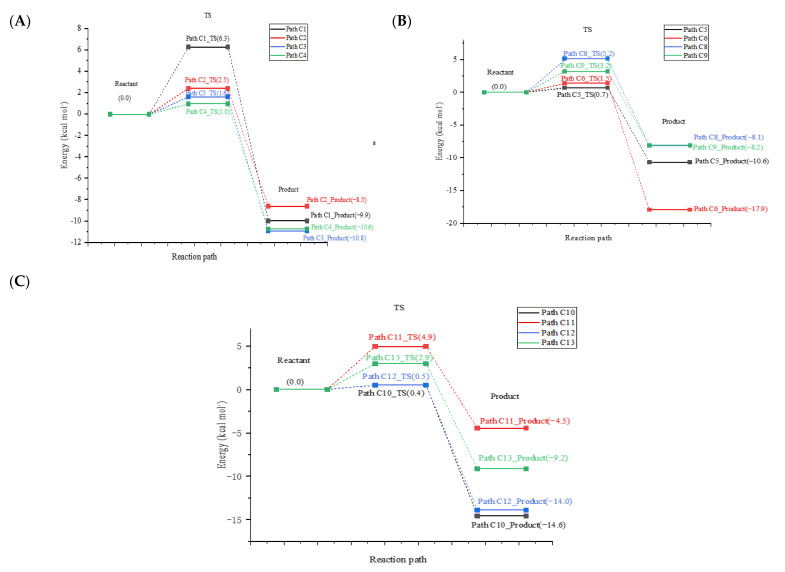
Potential energy surfaces for the reaction of CIT with ·OH at C1–C6/C8–C13. Sites along with computed activation energies (*E*_a_, kcal/mol) and enthalpy changes (Δ*H*, kcal/mol): (**A**) Path C1-Path C4; (**B**) Path C5-Path C6/Path C8-Path C9; (**C**) Path C10-Path C13.

The photochemical reaction of CIT by ·OH in water is shown in Figure 2. The benzene ring has six reaction sites, namely, C1–C6: C1, C2, C3, C4, C5, and C6. The reaction types of ·OH with the benzene ring at the C1–C5 sites are all addition reactions, and the substitution reaction of ·OH for F at the C6 site is shown in Figure 2. The *E*_a_ values of the reactions at the C1–C6 sites range from 0.7 to6.3 kcal/mol, and the corresponding reaction potential energy surfaces are shown in Figure 3. The reaction type of the C6 site is the substitution reaction of ·OH for F, and the corresponding reaction product has been detected in the experiment [29]. The NBO charge and spin density analyses revealed that the F produced during the photochemical process was a singly negatively charged anion. The order of *E*_a_ values corresponding to the six pathways in the reaction process of the six sites is C1 > C2 > C3 > C6 > C4 > C5. The corresponding *E*_a_ value for the C5 site addition reaction is the lowest, indicating that ·OH reacts most favorably with the C5 site in the reaction process of these six sites. It can be seen that both the addition and substitution at the C1–C6 sites are exothermic reactions with Δ*H* values ranging from –17.9 kcal/mol to –8.5 kcal/mol in Figure 3. The minimum Δ*H* value during the C6-site ·OH to F substitution reaction is –17.9 kcal/mol. The distances between the C1–C6 sites and ·OH in the corresponding TS state are 2.027–2.105 Å in Figures S1 and S2 (Supplementary Materials). It can be seen that the distance between ·OH and the reaction site during the substitution reaction is longer than that during the addition reaction. At the same time, the optimized geometries of R, TS, and P of each reaction of CIT with ·OH at the C1–C6 sites are shown in Figures S1 and S2 (Supplementary Materials).

The reaction paths of the six sites C8–C13 reacting with ·OH are shown in Figure 2. The reaction types corresponding to these six sites are all addition reactions. There are six places where the reaction can occur from C8 to C13, and the corresponding *E*_a_ values are 0.4–5.2 kcal/mol. It can be seen that the order of *E*_a_ values corresponding to the six sites is C8 > C11 > C9 > C13 > C12 > C10. The *E*_a_ value corresponding to the reaction at the C10 site was the lowest, indicating that the addition reaction at this site was the most favorable among the six sites. The reaction types of these six sites are all exothermic, and Δ*H* values range from –14.6 kcal/mol to –8.1 kcal/mol in Figure 3. It is obvious that the Δ*H* value of the C10 site reaction process is smaller than that of other sites, and it is concluded that the addition product at the C10 site is more stable than the products at the other five sites. The distances between the six sites of C8–C13 in the TS state and ·OH during the reaction are 1.945–2.113 Å in Figures S3 and S4. The optimized geometries of R, TS, and P for each reaction during the reaction of CIT with ·OH at the C8–C13 sites are shown in Figures S3 and S4 (Supplementary Materials).

The *E*_a_ values in the reaction process of the 12 sites corresponding to the two benzene rings are sorted based on the reaction sites as follows: C1 > C8 > C11 > C9 > C13 > C2 > C3 > C6 > C4 > C5 > C12 > C10. The *E*_a_ value corresponding to the C10 site is the lowest, and the reaction at this site is the most favorable of the 12 site reaction processes. The Δ*H* values for all reactions ranged from –17.9 kcal/mol to –4.5 kcal/mol, and all reactions were exothermic. The Δ*H* value was the smallest during the substitution reaction at the C6 site, indicating that the product of that substitution reaction was the most stable.

### 2.3. Indirect Photodegradation of CIT in Water via ^1^O_2_

**Figure 4:**
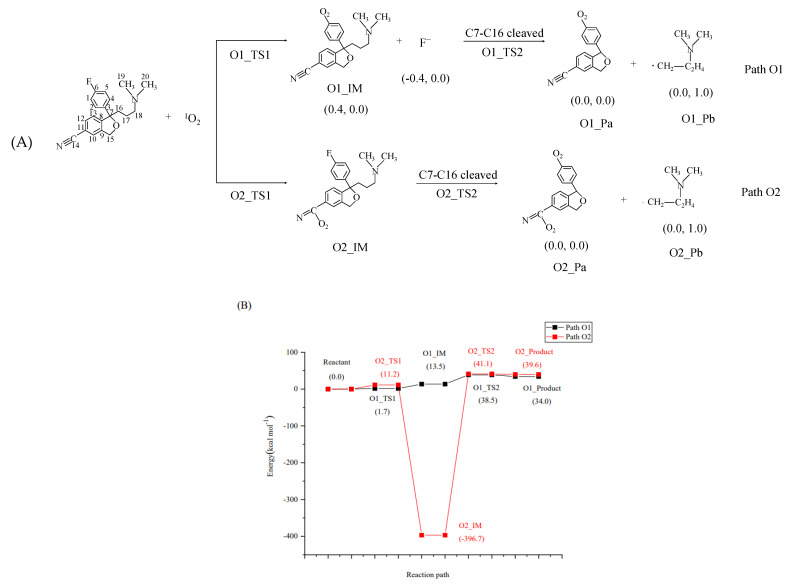
Indirect photodegradation pathways of CIT with ^1^O_2_, along with computed activation energies (*E*_a_, kcal/mol) and enthalpy changes (Δ*H*, kcal/mol). The figures in parentheses in Figure A are the NBO charge and electron spin density of the product: (**A**) Reaction pathway of CIT with ^1^O_2_; (**B**) Reaction potential energy surface of CIT with ^1^O_2_.

The photochemical reaction process between CIT and ^1^O_2_ is shown in Figure 4. Figures of the optimized TS, IM, and P are shown in Figures S5 and S6 (Supplementary Materials). The first step in the Path O1 reaction is the substitution of ^1^O_2_ for F at the C6 site. It can be seen that the *E*_a_ value of this process is 1.7 kcal/mol, and the Δ*H* value is 13.5 kcal/mol (Figure 4), indicating that this reaction is endothermic, and the substitution of ^1^O_2_ for F at the C6 site occurs easily. Through the analysis of NBO charge and spin density, it can be seen that the generated F is a singly negatively charged anion (Figure 4). O1_IM undergoes a C7–C16 cleavage reaction, and the *E*_a_ value of this process is 38.5 kcal/mol, which is significantly higher than that of ^1^O_2_ for the substitution reaction of F at the C6 site (Figure 4). This shows that the cracking reaction of C7–C16 is the key step in the whole process. The corresponding Δ*H* value is 34.0 kcal/mol, and the reaction type is endothermic. The product O1_Pb was a free radical with a spin density of 1.0 by NBO charge and spin density analyses, as shown in Figure 4.

^1^O_2_ undergoes an addition reaction with C at the C14 site to generate O2_IM. The corresponding *E*_a_ value in this reaction process is 11.2 kcal/mol, and the Δ*H* value is –396.7 kcal/mol in Figure 4. O2_IM undergoes a C7–C16 cleavage reaction to generate products O2_Pa and O2_Pb. The corresponding *E*_a_ value is 41.1 kcal/mol, and the Δ*H* value is 39.6 kcal/mol. The cleavage of C7–C16 is the key step in the overall reaction process. The NBO charge and spin density analyses of the product O2_Pb show that it is a free radical with a spin density of 1.0 in Figure 4. The C–O distance in the O1_TS1 state during the substitution reaction is 1.603 Å in Figure S5; the C–O distance in the O2_TS1 state is 1.553 Å during the addition reaction in Figure S6, and there is no significant difference between the two reactions. The C–O distances in O1_TS2 and O2_TS2 corresponding to the cleavage reaction of C7–C16 are 2.511 Å and 2.248 Å, respectively. At the same time, the cleavage reaction process of C7–C16 is the key step of Path O1 and Path O2.

### 2.4. Direct Photochemical Reaction of CIT in Water

**Figure 5:**
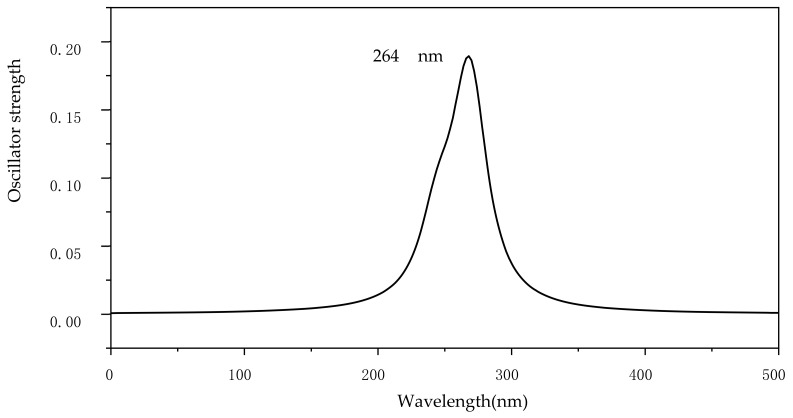
Calculated electronic absorption spectra of CIT with maximum absorption wavelength (nm).

**Figure 6:**
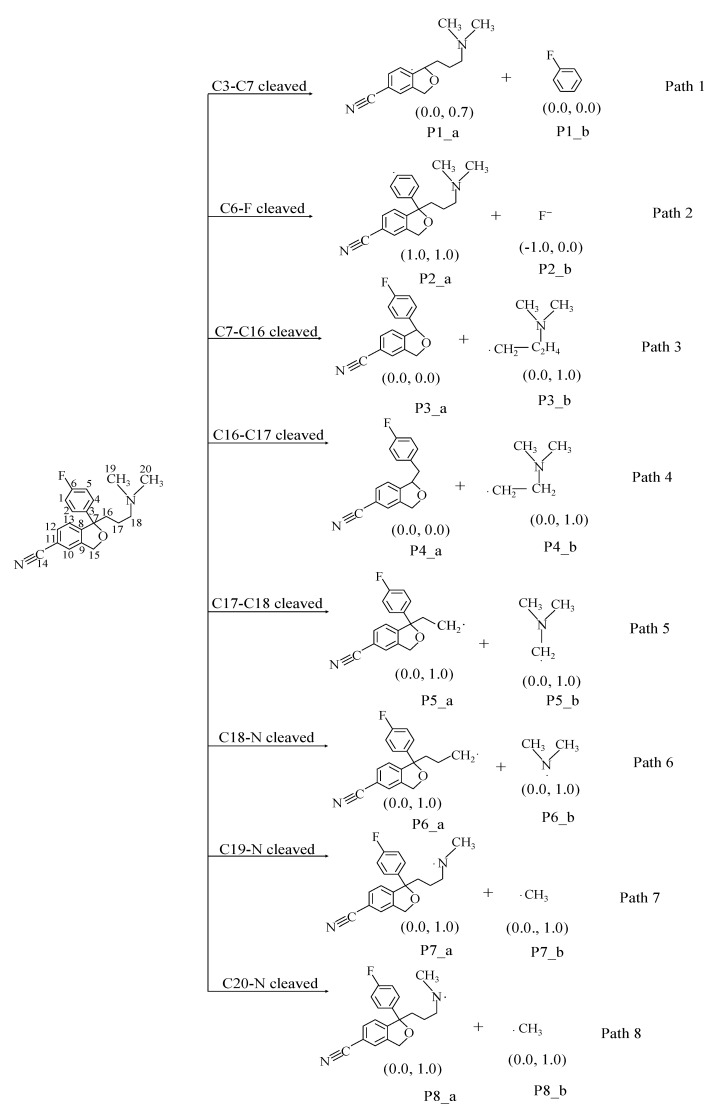
Direct photodegradation pathway of CIT. The figures in parentheses are NBO charge and electron spin density of products (NBO charge, electron spin density).

**Figure 7:**
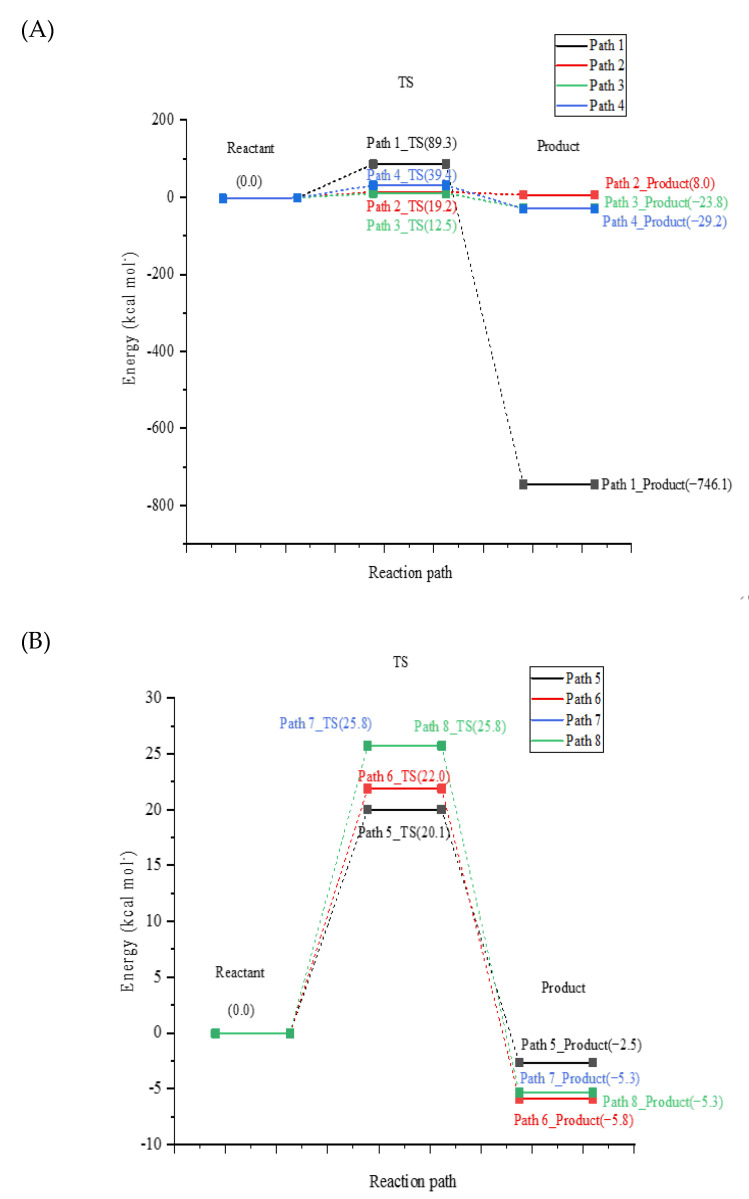
Potential energy surface for the direct photodegradation of CIT, along with computed activation energies (*E*_a_, kcal/mol) and enthalpy changes (Δ*H*, kcal/mol): (**A**). Path 1-Path 4; (**B**) Path 5- Path 8.

The calculated UV absorption spectrum of CIT is shown in Figure 5. The calculated electron absorption wavelength of CIT is about 264 nm, in accordance with the corresponding experimental value [38]. The corresponding eight reaction paths in the direct photodegradation process are shown in Figure 6. Except for the cleavage reaction of the C–F bond in Path 2, the cleavage reactions involve C–C bonds. The corresponding reaction potential energy surface graphs are shown in Figure 7. The corresponding *E*_a_ values in these eight reaction processes ranged from 89.3 to12.5 kcal/mol. The corresponding *E*_a_ value in Path 1 is the highest at 89.3 kcal/mol, so it is difficult to remove the benzene ring during direct photodegradation. The *E*_a_ value corresponding to the cleavage reaction of the C7–C16 bond in Path 3 is the lowest at 12.5 kcal/mol, so this reaction is the most likely to occur. The *E*_a_ values of the cleavage reactions of the C6–F/C17–C18/C18–N/C19–N/C20–N bonds all indicate that the corresponding photodegradation reaction could be carried out. The corresponding *E*_a_ value in the cleavage process of C16–C17 is 39.4 kcal/mol, which indicates that this pathway reaction is difficult to make happen. The Δ*H* values during the direct photodegradation ranged from –746.1 to 8.0 kcal/mol in Figure 7. The Δ*H* value during the C–F bond cleavage reaction was 8.0 kcal/mol, which was the only endothermic reaction during direct photodegradation. It was found that the *E*_a_ and Δ*H* values of C19–N and C20–N were consistent during the reaction.

The NBO charge and spin density analyses showed that P1_a was a free radical with a spin density of 0.7, and P3_b/P4_b/P5_a/P5_b/P6_a/P6_b/P7_a/P7_b/P8_a/P8_b were all free radicals with a spin density of 1.0. The product P2_a generated during the cleavage reaction of C6–F is a cationic radical with a spin density of 1.0, and P2_b is a singly negatively charged anion, which is the same as the experiment value [28]. The optimized geometries of TS, IM, and P during the direct photodegradation are shown in Figures S7 and S8 (Supplementary Materials). The C–C distances for cleavage in the TS are 1.960–2.406 Å, the C–F distance is 1.708 Å, and the C–N distances are 1.901–1.938 Å.

## 3. Computational Methods

CIT was calculated by Gaussian 09, which was a popular and widely used electronic structure calculating program and was demonstrated to have high accuracy and credibility. Gaussian 09 was developed by M. J. Frisch and his team. Based on the three fundamental laws (molecular orbital theory, valence bond theory, and density functional theory), we can quickly and reliably minimize the molecular structure, calculate the charge distribution of compounds, predict the transition state structure, and explore the reaction mechanism [39].

The efficiency and accuracy of DFT and TDDFT methods have been proven to be reliable methods for studying excited states of matter [39,40,41,42,43,44,45]. All the calculations in this paper are carried out in this theory. The B3LYP functional is used in the energy calculation in this paper, and the basis set is 6-311 + G(d,p) [39,40]. In the calculation, the solvent effect in water is considered and expressed in the form of the integral equation of the polarized continuum model (IEFPCM) [46]. All stationary points are determined by calculating the frequency of the geometry on the same horizontal plane. The absorption spectra of electrons in water were calculated by the above method, and the atomic charge and electron spin density were calculated by the natural bond orbital (NBO) method.

In the process of photochemical reactions, the lowest excited state (T_1_) is the precursor of the photochemical reactions of most compounds [47], so the reaction substance ®, transition state (TS), intermediate state (IM), and product (P) are optimized in the T_1_ state. The frequency was calculated to determine the existence of a unique virtual frequency for each TS. The internal response coordinates were calculated in the same way to connect each transition state to the corresponding reactants and products [48]. The activation energy (*E*_a_) and enthalpy change (Δ*H*) values during the reaction were calculated and the zero-point energy was corrected. All the above calculations were performed using the Gaussian 09 software package [38,49].

## 4. Conclusions

The photochemical reaction of CIT in water was studied in this paper. The calculated results show that there are eight reaction pathways in the direct photodegradation process, which can be generally divided into three categories: C–C bond cleavage reaction, C–F bond cleavage reaction, and C–N bond cleavage reaction. The cleavage reaction of the C7–C16 bond is the most likely to occur in the process of direct photodegradation by *E*_a_ value analysis. The calculation results of CIT in the indirect photodegradation process show that the reaction types of ·OH and CIT are divided into the addition reaction of ·OH and the substitution reaction of F. In the calculation of the hydroxyl group, the calculation results after the substitution of the hydroxyl group at the C6 site for F are consistent with the experimental results [29]. According to the calculated *E*_a_ value, the reaction of all 12 sites can occur, and the Δ*H* value indicates that the reaction between ·OH and CIT is exothermic. The reaction between ^1^O_2_ and CIT is divided into the substitution reaction of F and the addition reaction at the C14 site. C7–C16 cleavage will occur in the reaction of both pathways, and the calculation of the corresponding *E*_a_ value indicates that this step is difficult to make happen. Meanwhile, the cleavage reactions of C7–C16 are all endothermic reactions, and the generated products O1_Pb and O2_Pb are free radicals with a spin density of 1.0. In the process of direct photolysis calculation, the calculated C6-F bond cleavage value is consistent with the experimental conclusion [28]. Overall, the lowest activation energy of C10 was 0.4 kcal/mol during the reaction of the hydroxyl group. The *E*_a_ value of the process is 1.7 kcal/mol, which is the lowest activation energy required for the reaction of ^1^O_2_ with CIT. In the direct photodegradation of CIT, the activation energy of the C7-C16 cleavage reaction was the lowest, which was 12.5 kcal/mol. The ·OH-addition reaction and ·OH-substitution reaction, substitution reaction of ^1^O_2_ to F and addition reaction of ^1^O_2_ at the C14 site, as well as the cleavage reactions of C6–F/C7–C16/C17–C18/C18–N/C19–N/C20–N are the main pathways of CIT photodegradation. The above studies indicate that computational simulation can effectively explore the photodegradation process of antidepressant drugs in water.

## Data Availability

Not applicable.

## Supplementary Materials

The following supporting information can be downloaded at: https://www.mdpi.com/article/10.3390/molecules28124620/s1, Figure S1: Optimized geometries of reactant (R), transition state (TS), and product (P) at C1-C3 sites for the indirect photodegradation pathways of CIT with ·OH; Figure S2: Optimized geometries of reactant (R), transition state (TS), and product (P) at C4-C6 sites for the indirect photodegradation pathways of CIT with ·OH; Figure S3: Optimized geometries of reactant (R), transition state (TS), and product (P) at C8-C10 sites for the indirect photodegradation pathways of CIT with ·OH; Figure S4: Optimized geometries of reactant (R), transition state (TS), and product (P) at C11-C13 sites for the indirect photodegradation pathways of CIT with ·OH; Figure S5: Optimized geometries of reactant (R), transition state (TS), intermediate (IM), and product (P) in Path O1 for the indirect photodegradation pathways of CIT with ^1^O_2_; Figure S6: Optimized geometries of reactant (R), transition state (TS), intermediate (IM), and product (P) in Path O2 for the indirect photodegradation pathways of CIT with ^1^O_2_; Figure S7: Optimized geometries of reactant (R), transition state (TS), product (P) in C3-C7/C6-F/C7-C16/C16-C17 for the direct photodegradation pathways of CIT; Figure S8: Optimized geometries of reactant (R), transition state (TS), product (P) in C17-C18/C18-N/C19-N/C20-N for the direct photodegradation pathways of CIT.
