# Supplementary material for: Computational Study of Photodegradation Process and Conversion Products of the Antidepressant Citalopram in Water
Source: Molecules. 2023 Jun 7;28(12):4620. doi: 10.3390/molecules28124620 (PMC10304023; doi:10.3390/molecules28124620)
Supplement: Supplementary file 1 [file molecules-28-04620-s001.zip › molecules-2355672-supplementary.pdf]

Supplementary Material

Computational study of photodegradation process and conversion products of the antidepressant citalopram in water

Yifan Shen[a], Se Wang[a,*], Ying Lu[a], Kan Chen[a], Li Luo[b], Ce Hao[c]

[a] *School of Environmental Science and Engineering, Collaborative Innovation Center of Atmospheric Environment and Equipment Technology, Jiangsu Key Laboratory of Atmospheric Environment Monitoring and Pollution Control, Nanjing University of Information Science and Technology, Nanjing 210044, China*

[b] *School of Chemistry and Life Sciences, Suzhou University of Science and Technology, Suzhou, Jiangsu 215009, China*

[c] *State Key Laboratory of Fine Chemicals, Dalian University of Technology, Dalian, Liaoning 116024, China*

9 Pages

8 Figures

* Corresponding author. Tel./fax: +86 25 58731090. E-mail: wangse@nuist.edu.cn (S. W.)

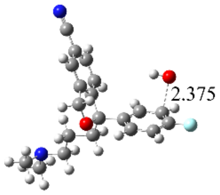

C1_R

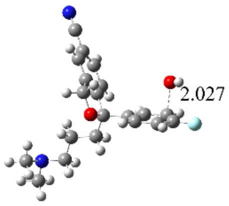

C1_TS

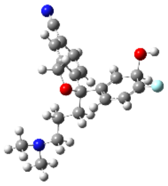

C1_P

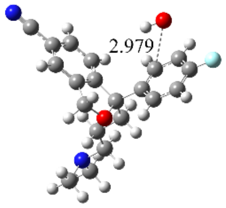

C2_R

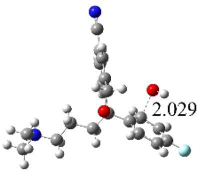

C2_TS

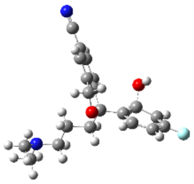

C2_P

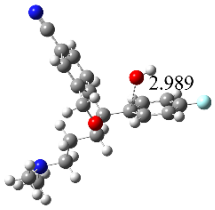

C3_R

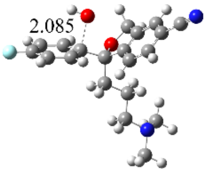

C3_TS

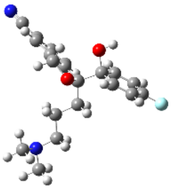

C3_P

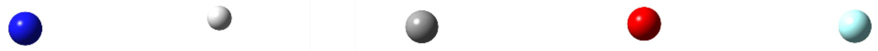

N H C O F

Figure S1. Optimized geometries of reactant (R), transition state (TS), and product (P) at C1-C3 sites for the indirect photodegradation pathways of CIT with ·OH.

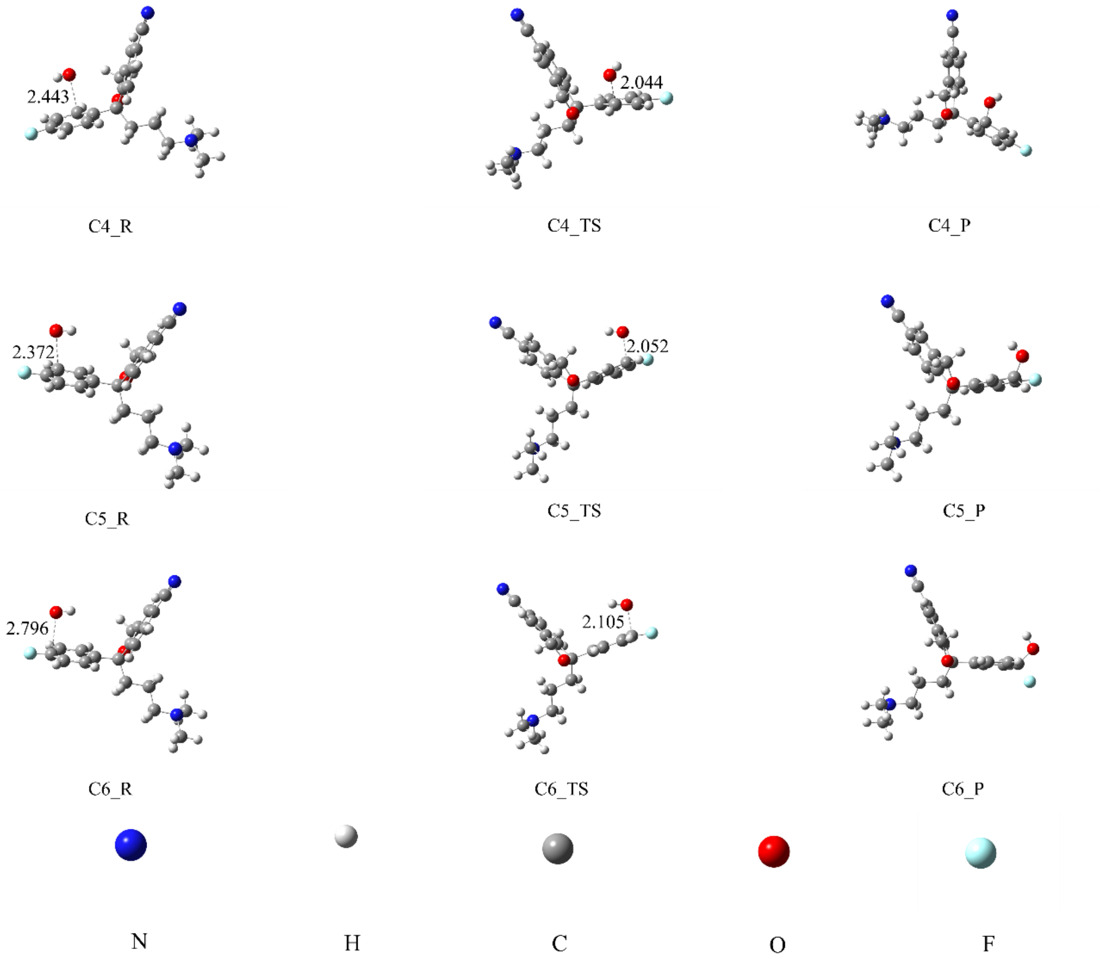

Figure S2. Optimized geometries of reactant (R), transition state (TS), and product (P) at C4-C6 sites for the indirect photodegradation pathways of CIT with ·OH.

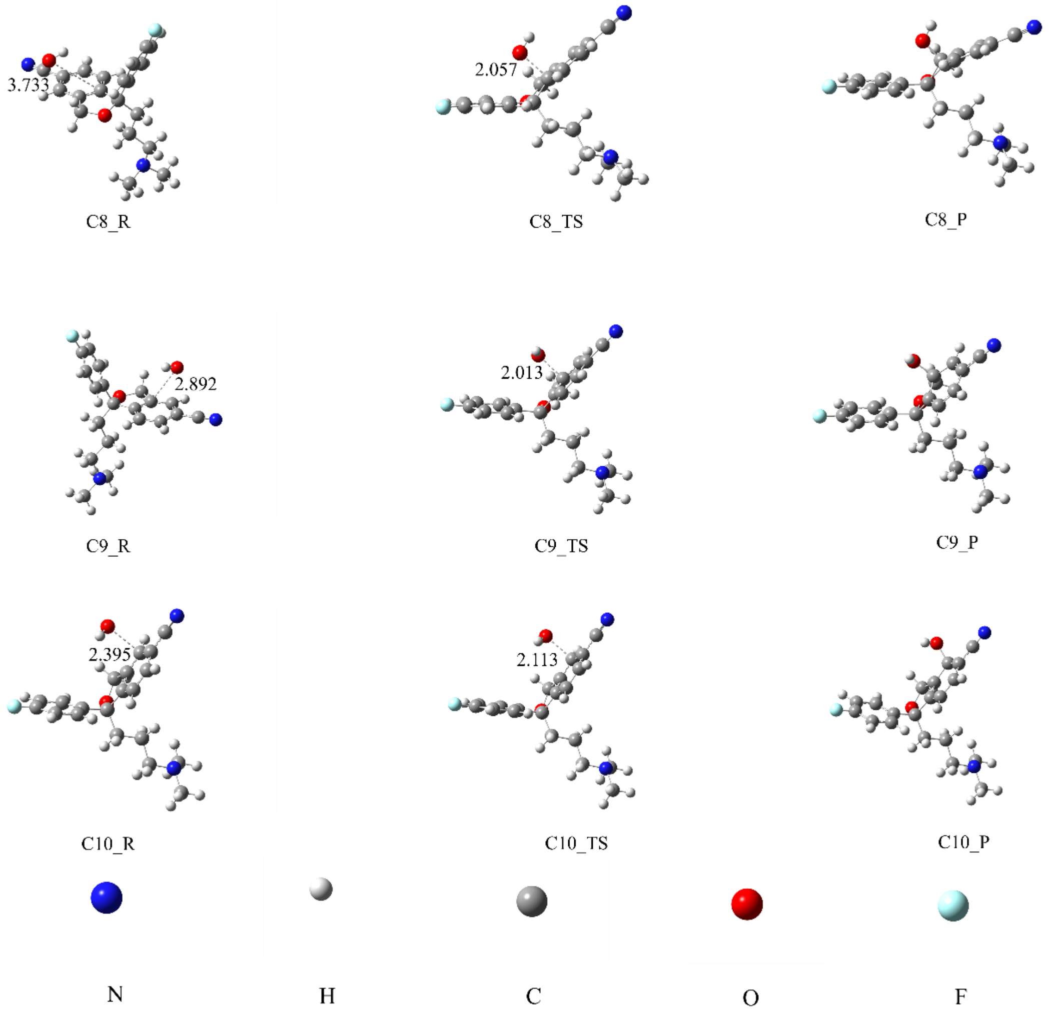

Figure S3. Optimized geometries of reactant (R), transition state (TS), and product (P) at C8-C10 sites for the indirect photodegradation pathways of CIT with ·OH.

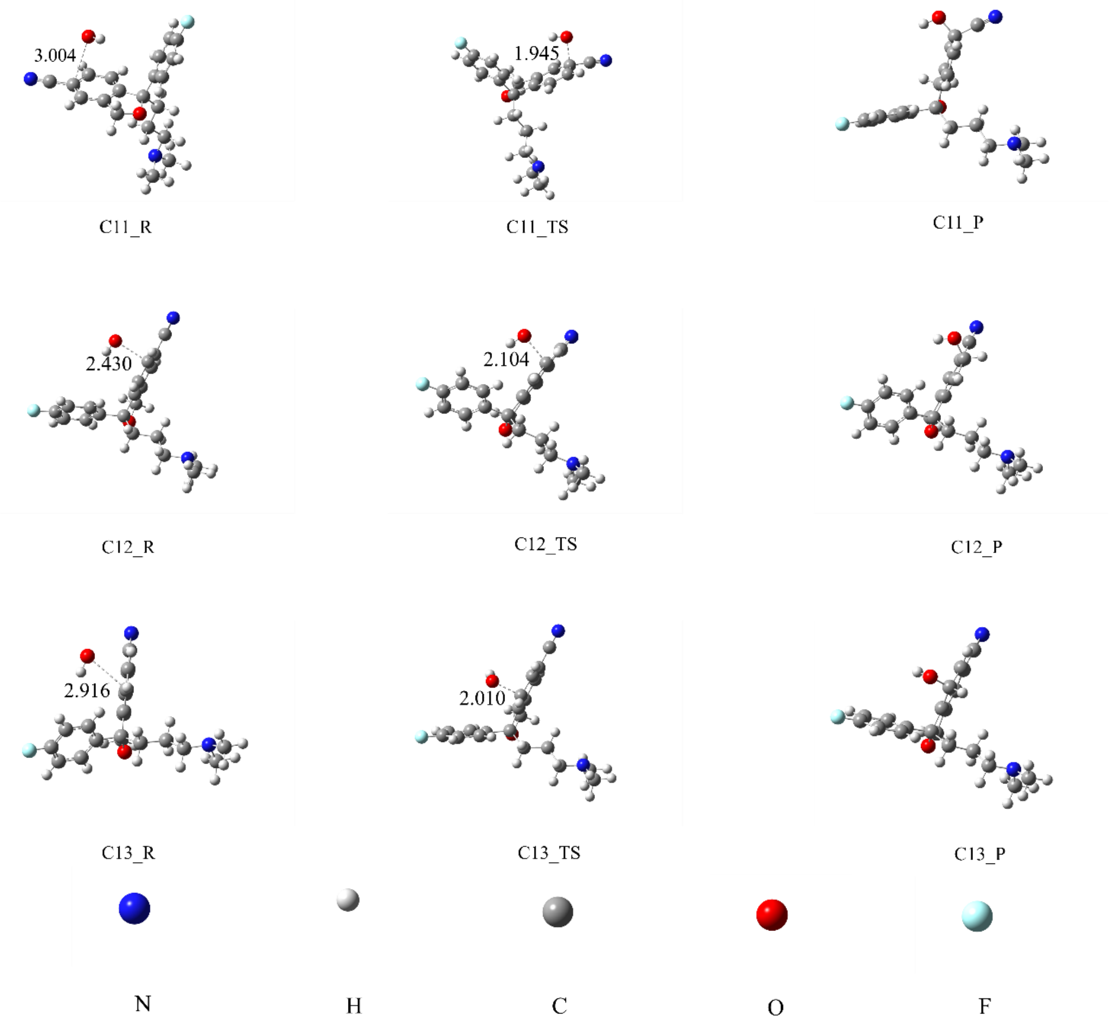

Figure S4. Optimized geometries of reactant (R), transition state (TS), and product (P) at C11-C13 sites for the indirect photodegradation pathways of CIT with ·OH.

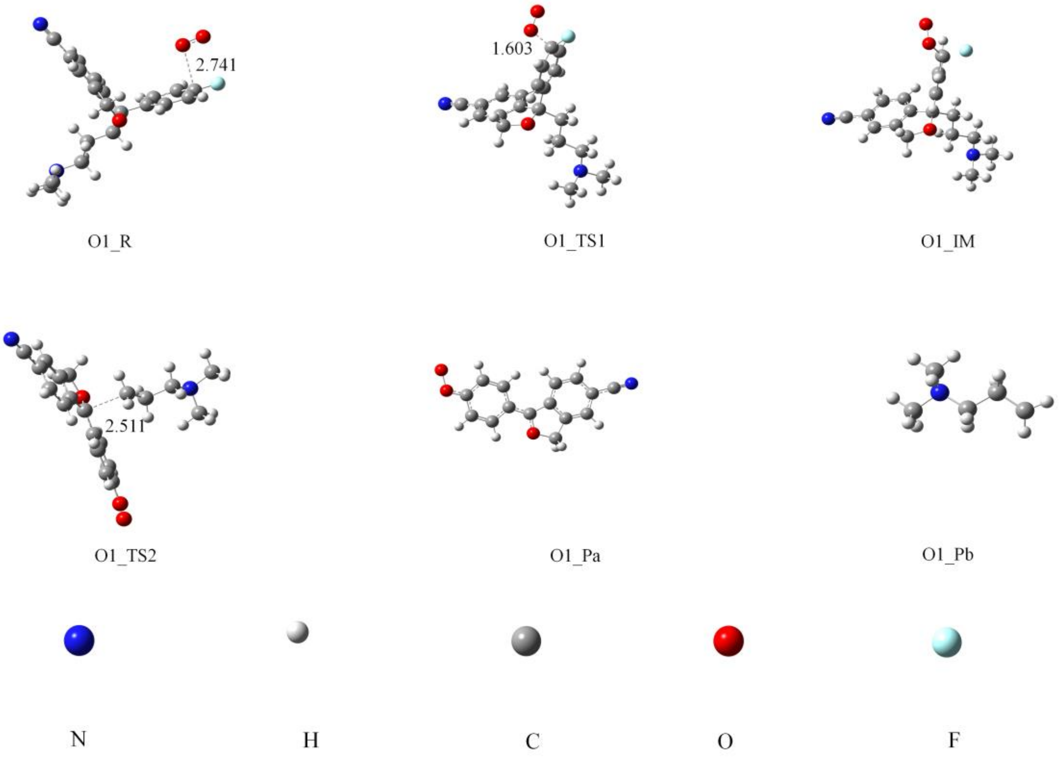

Figure S5. Optimized geometries of reactant (R), transition state (TS), intermediate (IM), and product (P) in Path O1 for the indirect photodegradation pathways of CIT with $^1O_2$.

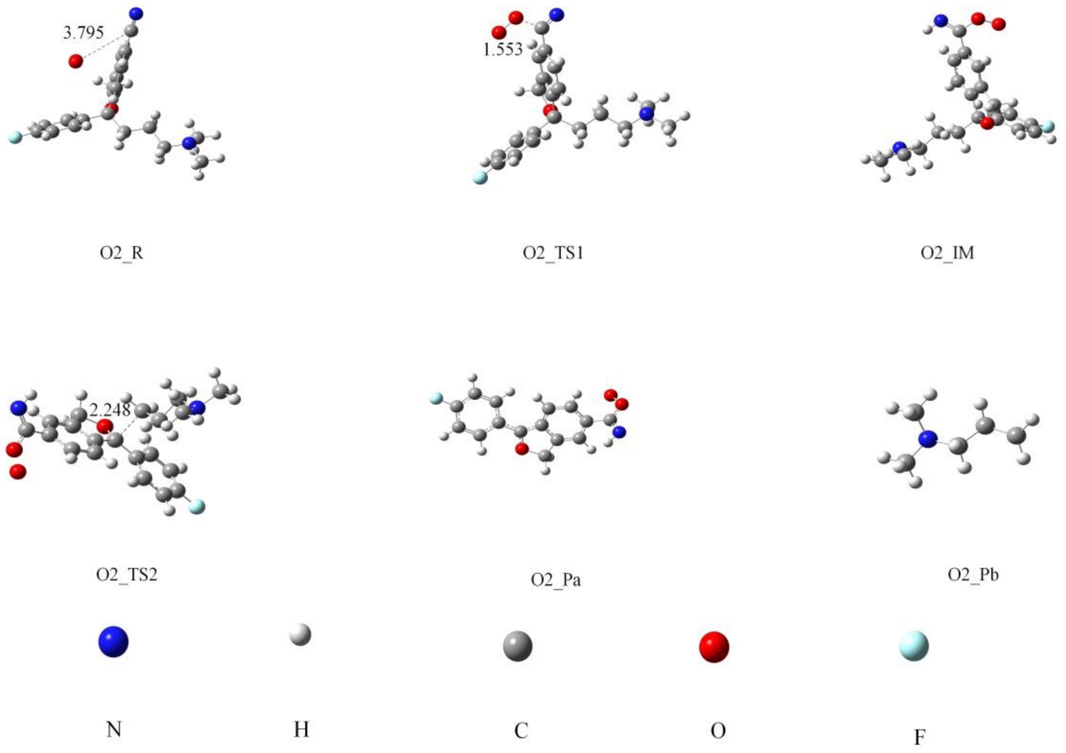

Figure S6. Optimized geometries of reactant (R), transition state (TS), intermediate (IM), and product (P) in Path O2 for the indirect photodegradation pathways of CIT with $^1O_2$.

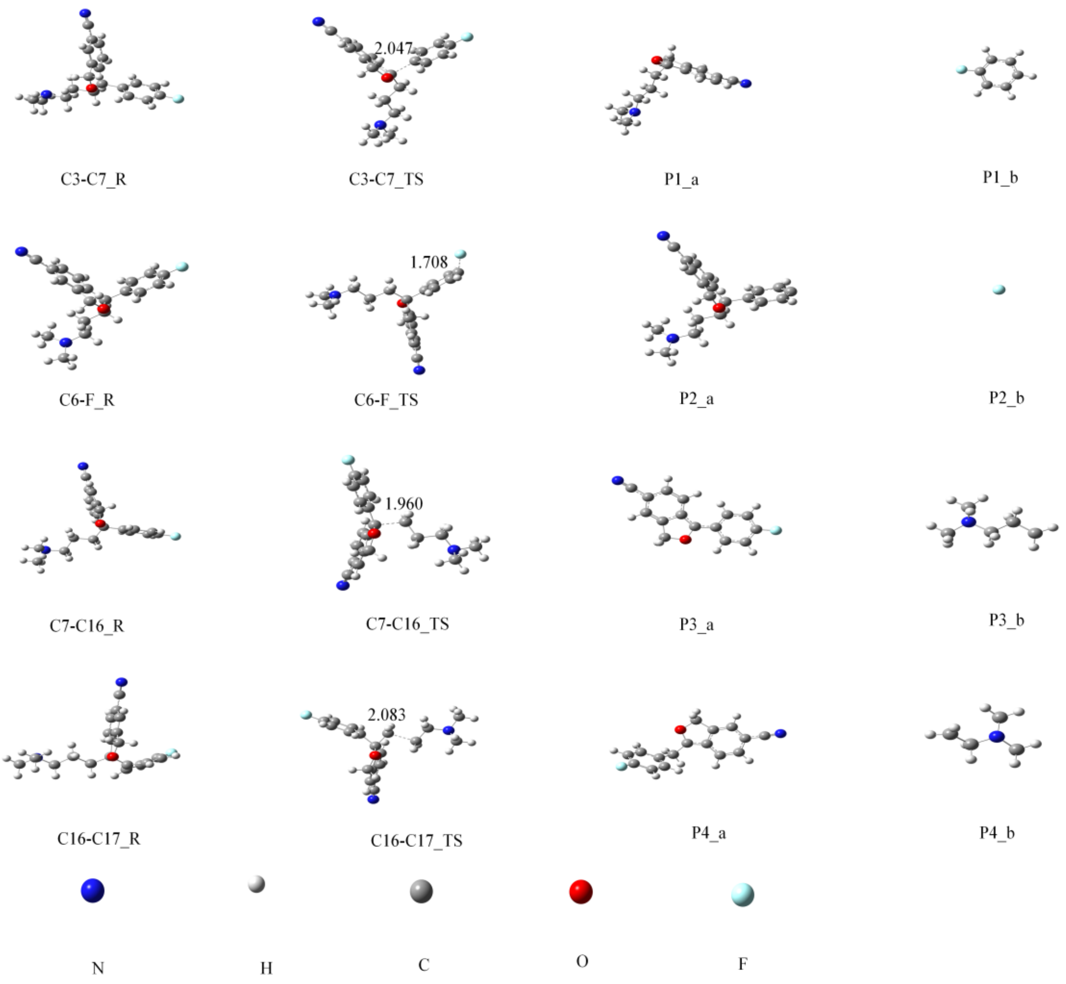

Figure S7. Optimized geometries of reactant (R), transition state (TS), product (P) in C3-C7/C6-F/C7-C16/C16-C17 for the direct photodegradation pathways of CIT.

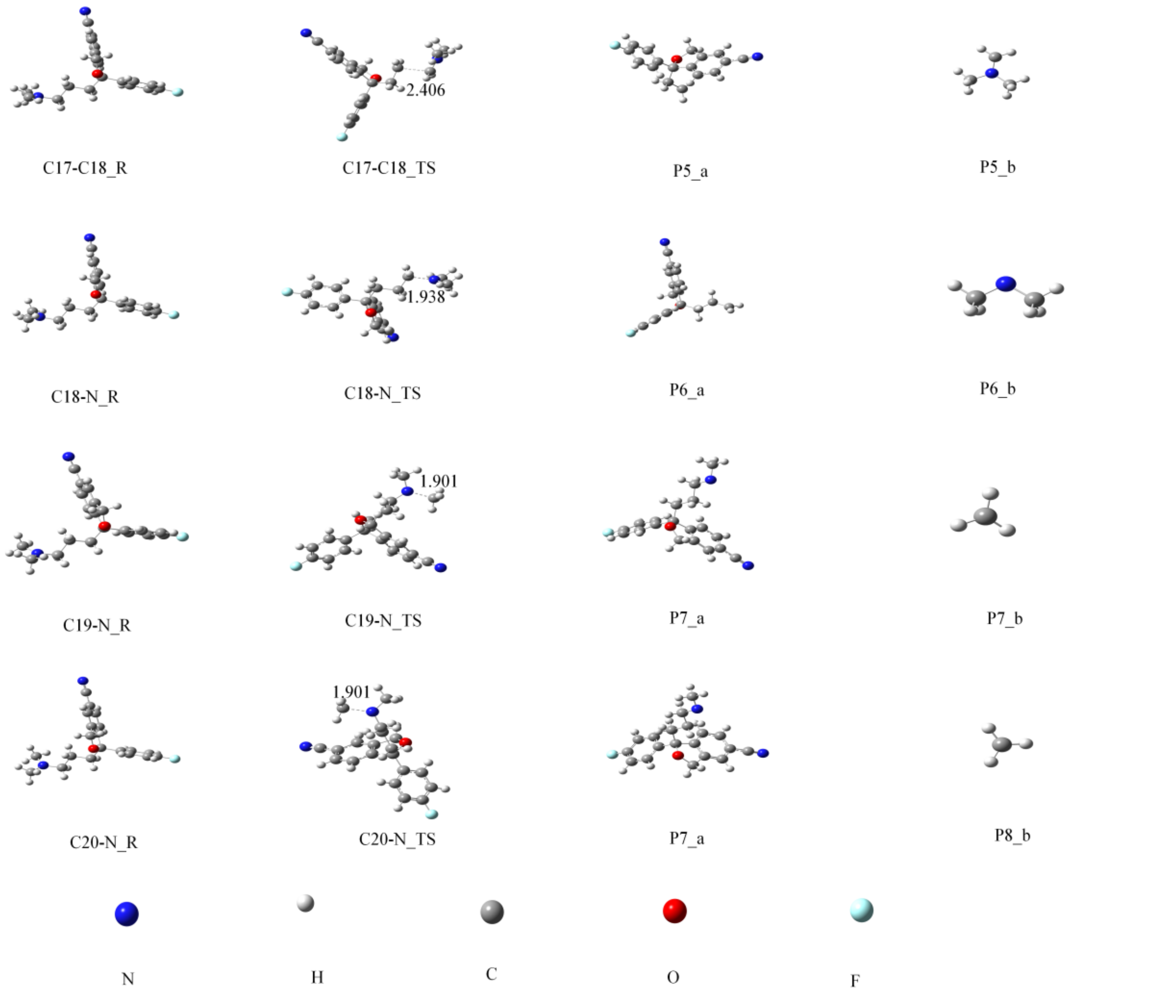

Figure S8. Optimized geometries of reactant (R), transition state (TS), product (P) in C17-C18/C18-N/C19-N/C20-N for the direct photodegradation pathways of CIT.
